# Teaching Ophthalmology for Machines

**DOI:** 10.2174/1874364101812010127

**Published:** 2018-06-29

**Authors:** Thiago Gonçalves dos Santos Martins, Maria Carolina Francisco Kuba, Thomaz Gonçalves dos Santos Martins

**Affiliations:** 1 Federal University of São Paulo São Paulo Brazil; 2 Casa de Portugal Portugal; 3 Piedade Hospital Rio de Janeiro Brazil

Ophthalmologic care is scarce in many parts of the world. Without early diagnosis and timely treatment, patients with ophthalmological diseases eventually reach advanced stages of the disease, such as blindness.

Ophthalmology teaching in medical schools has been reduced over time. For example, in the United States, the compulsory study of ophthalmology in medical schools decreased from 68% in 2000 to 30% in 2004 [1].

It is expected that all doctors and nurses should know about pediatrics and internal medicine. The same does not apply to ophthalmology. The same could be true for nursing. Approximately 98% of medical doctors will not be the specialists in ophthalmology which makes it important for all physicians to learn basic ophthalmology [2].

In 1996, a survey conducted in Canada found that more than half of the curricula of medical schools in that country did not have a mandatory internship in ophthalmology [2]. In the United Kingdom, the average duration of medical curriculum of ophthalmology is 7.6 days, with 21% of medical schools not having compulsory education in ophthalmology [3]. The same could be true for nursing curriculum. Therefore, the resolution of all ophthalmological problems is concentrated in about 2% of trained physicians. In some countries, there is an aggravating factor including the unequal distribution of the medical doctors within the territory [4].

Physicians and engineers are currently working together to improve early ophthalmology diagnosis and follow-up. Algorithms are created for what is being called machine learning to assist medical decision-making and improve medical care. With the aim of providing better health service to populations, research has been done to develop new protocols of care that involve the use of artificial intelligence as a new tool for physicians to diagnose their patients more effectively and quickly [5].

Algorithms are simple commands for computers to make their own decisions. In order to develop the algorithms, a multidisciplinary team of doctors, engineers and mathematicians determine how computers can be useful for medical diagnosis by considering a huge database provided by physicians. The research for the development of such algorithms is already advanced in the area of oncology with the use of Watson, an IBM supercomputer that is able to use and interpret data generating hypotheses. Most importantly, this technology provides the opportunity to learn, aiding medical doctors in decision-making processes. Machine learning classifiers considered as input empirical data predicted the features of the data. Machine learning has been applied to speed the process of testing hypothesis. It uses an extensible set of structured and unstructured content sources as well as broad range of pluggable search and scoring components that allow integration of many different analytic techniques. Machine-learning is used to learn the weights for combining scores from different scorers. This analysis produces hundreds of features. These features are then combined based on their learned potential for predicting the right answer. Improving diagnostic accuracy and speed can directly improve quality of care in patients as well as reduce the overall cost incurred in this process by health care systems. Watson will assist healthcare practitioners in evaluating a set of hypotheses [6].

In ophthalmology, research in the area of ​​artificial intelligence seeks to assist the health care professional in 1) screening of ophthalmological diseases in regions lacking specialized professionals and 2) follow-up of chronic diseases. Computers have been programmed to compare images of patients with chronic diseases such as glaucoma and diabetes that can lead to blindness if not diagnosed and treated properly. IBM Watson has used the machine learning technique with the development of algorithms to identify signs of bleeding in retinal photographs of patients with diabetic retinopathy, which are captured and analyzed by a “neural” network created for image recognition and analysis. A neural network is composed of multiple layers of nodes: an input layer, one or more hidden layers, and an output layer. The computer will improve its performance from new images that are provided for analysis. Researchers report that the computer spends 20 seconds to analyze the image and has an accuracy of 86% in its severity rating of diabetic retinopathy [7]. In the future, this technology will enable the patient to be referred early to the ophthalmologist and assist in the remote monitoring of patients. Recently, there have been several studies reporting on deep learning algorithms in development for the detection of diabetic retinopathy. The use of deep learning in the treatment of diabetes and important since 30 to 40% of patients do not adhere properly to treatment [7]. Another important use of algorithms in ophthalmology was for the use of IOL in cataract lenses, in order to increase its accuracy [8]. Another example in ophthalmology was the development of the Retmaker for the follow-up of diabetic retinopathy. The investigators found that the Retmarker achieved acceptable sensitivity for referable retinopathy compared with manual graders while being more costeffective options [9].

The algorithms are not for the purpose of contributing to but not replacing the physicians' decision making. In addition, with the technique of machine learning, the computers themselves begin to learn from their accumulated experience as well as their users. The learning machines are already used in our daily lives. The suggestion of a movie to watch or a book to buy is based on previous research that taught the machine about our preferences. That way, the computer is already able to single-handedly profile our preferences. As seen earlier, this same technique is very useful in health research, helping doctors make decisions based on large databases (Big Data) given by the computer. This is a small step towards the construction of intelligent machines, but they are already capable of learning from new experiences, which is a fact that is to be taken into account in the medical education of future physicians, who can add this interaction with the machine to their academic education.

The first person to develop algorithms was Euclides, in century III A. C., which was long before the invention of the computer. It was developed in his work of The Elements (300 BC) as a simple and efficient method of finding the greatest Maximal Common Divisor (MCD) between two non-zero integers. The MCD of two integers is the largest integer that divides both without a remainder. The Euclidean algorithm is based on the principle that the MCD does not change if the smallest number is subtracted from the largest [8-10].

For a long time, algorithms were created by mathematicians for mathematicians. Now they are created for computers with the assistance of multidisciplinary professionals, such as in the health field, who seek to facilitate and improve medical care in regions where there is an uneven distribution of physicians and ophthalmology team members [11].
